# An Experimental Analysis of the Molecular Effects of Trastuzumab (Herceptin) and Fulvestrant (Falsodex), as Single Agents or in Combination, on Human HR+/HER2+ Breast Cancer Cell Lines and Mouse Tumor Xenografts

**DOI:** 10.1371/journal.pone.0168960

**Published:** 2017-01-03

**Authors:** Qing Chen, Ziyi Weng, Yunshu Lu, Yijun Jia, Longlong Ding, Fang Bai, Meixin Ge, Qing Lin, Kejin Wu

**Affiliations:** 1 Department of General Surgery, XinHua Hospital, Shanghai Jiao Tong University School of Medicine, Shanghai, China; 2 Department of General Surgery, Shanghai International Medical Center, Shanghai, China; 3 Department of Radiation Oncology, The Shanghai Tenth People's Hospital of Tongji University, Shanghai, China; 4 Department of Breast Surgery, Obstetrics and Gynecology Hospital of Fudan University, Shanghai, China; Taipei Medical University, TAIWAN

## Abstract

**Purpose:**

To investigate the effects of trastuzumab (herceptin) and fulvestrant (falsodex) either in combination or alone, on downstream cell signaling pathways in lab-cultured human HR+/HER2+ breast cancer cell lines ZR-75-1 and BT-474, as well as on protein expression levels in mouse xenograft tissue.

**Methods:**

Cells were cultivated in the presence of trastuzumab or fulvestrant or both. Molecular events that resulted in an inhibition of cell proliferation and cell cycle progression or in an increased rate of apoptosis were studied. The distribution and abundance of the proteins p-Akt and p-Erk expressed in these cells in response to single agents or combinatorial treatment were also investigated. In addition, the effects of trastuzumab and fulvestrant, either as single agents or in combination on tumor growth as well as on expression of the protein p-MED1 expressed in *in vivo* mouse xenograft models was also examined.

**Results:**

Cell proliferation was increasingly inhibited by trastuzumab or fulvestrant or both, with a CI<1 and DRI>1 in both human cell lines. The rate of apoptosis increased only in the BT-474 cell line and not in the ZR-75-1 cell line upon treatment with fulvestrant and not trastuzumab as a single agent (P<0.05). Interestingly, fulvestrant, in combination with trastuzumab, did not significantly alter the rate of apoptosis (in comparison with fulvestrant alone), in the BT-474 cell line (P>0.05). Cell accumulation in the G1 phase of cell cycle was investigated in all treatment groups (P<0.05), and the combination of trastuzumab and fulvestrant reversed the effects of fulvestrant alone on p-Akt and p-Erk protein expression levels. Using ZR-75-1 or BT-474 to generate *in vivo* tumor xenografts in BALB/c athymic mouse models, we showed that a combination of both drugs resulted in a stronger inhibition of tumor growth (P<0.05) and a greater decrease in the levels of activated MED1 (p-MED1) expressed in tumor issues compared with the use of either drug as a single agent.

**Conclusions:**

We demonstrate that the administration of trastuzumab and fulvestrant in combination results in positive synergistic effects on both, ZR-75-1 and BT-474 cell lines. This combinatorial approach is likely to reduce physiological side effects of both drugs, thus providing a theoretical basis for the use of such combination treatment in order to resolve HR+/HER2+ triple positive breast cancer that has previously been shown to be resistant to endocrine treatment alone.

## Introduction

In the last few decades, individualized treatment has played a significant role in the management of breast cancer patients. Such interventions, focused on targeting specific biological features of tumors, constitute a very effective strategy for the resolution of malignancies. The human epithelial growth factor receptor 2 (HER2) oncoprotein, along with the hormone receptors (HR) estrogen receptor (ER) and progesterone receptor (PR), are mediators of two key pathways involved in breast carcinogenesis, invasive behavior and cell growth, and have previously been validated as therapeutic targets[1,2].

Breast cancer is a molecularly heterogenous disease and several different sub-types have been defined based on cell receptor expression profiles. Approximately 25% of all female breast cancers exhibit an over-expression of HER2, which is known to drive aggressive cellular behavior[3–7]. Trastuzumab (a monoclonal antibody), the first-line of treatment for HER2+ breast cancers[8–10], has been shown to be active as a single agent[11,12] as well as in combination with chemotherapy[9,10,13] for the treatment of advanced stage HER2+ breast cancer. Its use has been shown to positively affect patient outcomes such as progression-free survival (PFS) and overall survival (OS). HR signaling pathways play an equally important role in breast cancer oncogenesis and advancement[1,2]. HR+ breast cancers account for about 70% of all invasive female breast malignancies and generally respond well to endocrine therapy[1,7]. However, side effects such as resistance to either HER2-targeted therapy or hormonal therapy along with other issues such as an increased cardiotoxicity caused by trastuzumab represent pressing clinical issues that pertain to the use of these drugs and the mechanisms for primary or acquired resistance to such therapies are probably multifactorial[1,14–17].

Previous literature has demonstrated that the targeted treatment of HER2+ breast tumors is associated with an increased resistance to endocrine treatment[18]. Since triple positive HR+/HER2+ breast cancer patients tend to be less responsive to endocrine therapy[19], we propose that extensive interactions exist between the HER2 signaling pathway and the ER signaling pathways and a large body of recent literature supports this hypothesis[19–23]. Studies report that once growth stimuli (including ligands), bind to the HER2 receptor, a series of intracellular signaling pathways are activated. These mainly include the PI3K/Akt/mTOR and MAPK/ERK signal pathways, downregulating ER expression via phosphorylation of the PI3K/Akt/mTOR signal pathway and phosphorylating ER Ser118 via activation of the MAPK/ERK pathway, which correlates with the poor clinical outcomes of breast cancers[2,22,24,25]. Our study might enable the identification of the possible mechanism by which HER2 expression participates in the resistance to endocrine treatment.

In addition, the ER co-activator Mediator Subunit 1 (MED1) has been found to be a novel critical cross-talk point for the HER2 and ER signaling pathways within the cell and is amplified or overexpressed in more than 50% of all primary breast cancers[26,27]. It has been demonstrated that the MED1 gene, localized on chromosome 17q12, is required for ER-dependent endogenous reporter gene expression and estrogen-dependent breast cancer cell growth[28–32]. Certain studies have reported that MED1 amplification or overexpression is significantly associated with poor outcomes in breast cancer patients treated with hormone therapy[33]. It also plays a critical role in the development of tamoxifen resistance, which is in turn linked with HER2 amplification or overexpression[30]. Experiments (both *in vitro* and *in vivo*) have demonstrated that a knockdown of MED1 sensitizes breast cancer cells to fulvestrant treatment[27]. Much literature has confirmed that the ER co-activator MED1 often co-amplifies with HER2 in primary breast cancers[26,27] and that HER2 amplification or overexpression can enhance MED1 phosphorylation by activating the MAPK/ERK pathway, thus affecting ER activity[34–36].

Data from clinical studies has shown that the majority of HR+/HER2+ breast cancer patients are minimally responsive to endocrine therapy and it is also well known that HER2 positivity plays a critical role in the resistance to hormonal therapy[18,19]. In addition, the cardiotoxic effects of trastuzumab such as decreased left ventricular ejection fraction, congestive heart failure and even death are commonly encountered in routine clinical practice[37,38]. As reported by C. Kent Osborne in the American Society of Clinical Oncology (ASCO) Annual Meeting on June 4 2016, studies on crosstalk between estrogen and growth factor receptor pathways may reveal new strategies to overcome endocrine therapy resistance. Therefore, targeting the ER and HER2 receptors simultaneously is likely to be a fruitful approach for the treatment of HR+/HER2+ (triple positive) breast cancer, and the occurrence of side effects may decrease significantly in this combination application. As fulvestrant, a selective ER down-regulator, remains active in HER2+ breast cancer cells that are resistant to other endocrine treatments and is licensed for advanced breast cancer in postmenopausal women after recurrence or progression despite previous endocrine treatments, it is possible that HER2 amplification or overexpression may have a different impact upon the activity of fulvestrant compared with tamoxifen or other hormone-regulating drugs[39–42]. Therefore, in this work, we test the effects of trastuzumab (herceptin) and fulvestrant (falsodex) as a combination in the HR+/HER2+ breast cancer cell lines ZR-75-1 and BT-474 as well as in mouse tumor xenografts.

## Materials and Methods

### Drugs and Cells

Trastuzumab (herceptin) and fulvestrant (falsodex) were purchased from Roche (Basel, Switzerland) and AstraZeneca (London, England), respectively, and stored at 4°C according to manufacturer’s instructions. The HR+/HER2+ breast cancer cell lines ZR-75-1 and BT-474 were obtained from the Cell Bank of the Chinese Academy of Sciences (Shanghai, China) and the Cell Resource Center (Beijing, China), respectively. Cells were cultured in RPMI 1640 supplemented with 10% fetal bovine serum (FBS) and 1% penicillin-streptomycin (PS) in a 37°C, 5% CO_2_ fully humidified incubator. In addition, 0.01mg/ml of insulin was added in order to culture BT-474 cells.

### Ethical Considerations

Nude mice were used as animal models in order to study the effects of trastuzumab combined with fulvestrant in HR+/HER2+ breast cancer. All experiments were carried out in strict accordance with the recommendations of the Guide for the Care and Use of Laboratory Animals (National Institutes of Health). The protocol was approved by the Committee for the Ethics of Animal Experiments of the Xinhua Hospital, affiliated to Shanghai Jiao Tong University School of Medicine, China. Surgeries were performed using sodium pentobarbital as an anesthetic and animals were sacrificed by CO_2_ asphyxiation. Every effort was made in order to minimize animal suffering.

### Cell Proliferation Inhibition Rate

ZR-75-1 cells or BT-474 cells were seeded in 96-well plates at a density of 2.5^10^4^ cells/100μl culture/well and cultivated in medium containing phenol red-free RPMI 1640. After they adhered successfully, cells were treated with suspensions containing the following components, viz., no drug, a vehicular control, different concentrations of trastuzumab, different concentrations of fulvestrant or various combinations of the two. After 72 hours, cells were incubated in culture supplemented with 10% WST-1 (Roche, USA) for 2.5h in a 37°C incubator. Absorbance (A) at 460nm was detected and cell proliferation inhibition rate (CPIR) was calculated using the following formula: CPIR = 1-(A_treated_-A_blank_)/(A_control_-A_blank_), where A_treated_, A_blank_ and A_control_ represent the cell absorbance in the treated group, blank group (no cells plated) and control group (cells cultured with no drugs) respectively.

### Analysis of Synergy

Data from the CPIR assay was analyzed using the CalcuSyn software. Combination index (CI) and dose-reduction index (DRI) at a 50% inhibition effect level were calculated from the effects of varying doses on cell proliferation inhibition rates. CI is a quantitative measure of the degree of drug interaction and may be classified in terms of additive effect (CI = 1), synergism (CI < 1), or antagonism (CI > 1) for a given endpoint[43]. DRI is a measure of how much the dose of each drug in a synergistic combination may be reduced at a given effect level compared with doses of each drug when used as single agents.

### Analysis of Apoptotic Rate by Flow Cytometry

ZR-75-1 and BT-474 cells (in the exponential growth phase) were seeded in 6-well plates. After the cells adhered to the bottom of the plates, they were either left untreated, treated with a vehicular control, trastuzumab at 0.65mg/ml in ZR-75-1 cells and 0.13mg/ml in BT-474 cells, or fulvestrant at 45nmol/L in ZR-75-1 cells and 34nmol/L in BT-474 cells, or a combination of trastuzumab at 0.65mg/ml and fulvestrant at 45nmol/L in ZR-75-1 cells or trastuzumab at 0.13mg/ml and fulvestrant at 34nmol/L in BT-474 cells. After 72h, cells were collected and stained with annexin V-fluorescein isothiocyanate (Annexin V-FITC) and propidium iodide (PI) using the annexin V-FITC/PI apoptosis detection kit (BD Pharmingen; San Diego, CA, USA) in accordance with manufacturer’s instructions. Flow cytometric analysis was performed using the BD FACS Calibur Cell Sorting System (BD Pharmingen; San Diego, CA, USA) and results were analyzed using the FlowJo 7.6.1 software.

### Analysis of Cell Cycle progression by Flow Cytometry

After treatment with the same concentrations and combinations of both drugs as used in previous experiments, cells were collected, washed and re-suspended in ice-cold PBS and fixed using 70% ethanol at 4°C for over 24h. Thereafter, cells were centrifuged, re-suspended in ice cold PBS and then stained with PI using the cell cycle and apoptosis analysis kit (Beyotime Biotechnology, China) at 37°C for 30 minutes. Cells were then detected using the BD FACSCalibur CellSorting System and results were analyzed using FlowJo 7.6.1 software.

### Analysis of Protein Expression by Immunoblotting

Three days after treatment, cell proteins in all groups were extracted separately. Protein samples (50μg) were separated on a 10% SDS-PAGE gel and then transferred to a PVDF membrane. Subsequently, membranes were blocked using BSA. This was followed by incubation in primary antibodies at 1:1000 dilution in TBS-Tween (TBST) (0.05% Tween-20 in TBS) at 4°C overnight on a shaker. Anti-phospho-Akt (Ser-473) (#4060), anti-phospho-p44/42 MAPK (Erk1/2) (Thr 202/Try 204) (#4370), anti-Akt (#9272), anti-p44/42 MAPK (Erk1/2) (#9102) and anti-GAPDH (#2118) antibodies were purchased from Cell Signaling Technology (USA). Membranes were then incubated in secondary peroxidase-conjugated antibody dilution buffer (Cell Signaling Technology, USA) and processed using immobilon western chemiluminescence HRP substrate kit (Millipore, USA).

### Mouse Xenograft Studies

4-5-week-old female BALB/c (nu/nu) athymic nude mice were purchased from Shanghai Animal Center (Shanghai, China) and maintained in sterilized cages with filtered air, 12h light-dark cycle, and sufficient sterile water and food in the Xin Hua Hospital Animal Center (Shanghai, China). 1 week post acclimatization, ZR-75-1 (1x10^7^ cells) or BT-474 (2.5x10^7^ cells) breast cancer cells respectively were re-suspended in 100μl or 200μl mixture of 50%PBS/50% Matrigel (BD Biosciences) which was injected subcutaneously into both sides of the mammary fat pad in the flank region of the mice. Six weeks later, animals bearing ZR-51 tumors reaching 150 to 250 mm^3^ in size were selected and randomly assigned to four groups receiving one of the following therapies, viz, PBS (control), trastuzumab, fulvestrant or both. After an additional two weeks, most BT-474 tumors grew up to 150 to 250 mm^3^ and they were randomly allocated to the four different experimental groups. Subsequently, trastuzumab at 2.5mg/kg or 2.0mg/kg body weight was injected intraperitoneally (i.p.) twice a week into mice bearing ZR-75-1 or BT-474 cells in the trastuzumab-treated and combination groups, respectively. Fulvestrant at 4mg/body or 2.5mg/body was administrated intramuscularly (i.m.) once a week to animals bearing ZR-75-1 or BT-474 cells for four weeks in the fulvestrant-treated and combination groups. Besides, animals in control group were treated i.p. and i.m. with the same volume of PBS as the volume of drugs administrated to mice in the combination groups. Tumors were measured twice a week and mice were weighted once a week. Tumor volume was calculated as (D x d^2^)/2 where D and d respectively represent the largest and smallest diameters. After four-week treatments, tumors were excised from mice under sodium pentobarbital anesthesia and stored at -80°C for immunoblot analysis. Subsequently, animals were sacrificed by CO_2_ asphyxiation.

### Immunoblot Analysis of Proteins p-MED1 and MED1 Expressed in Tumor Tissues

Frozen tumor tissues were ground and lysed in RIPA buffer on ice until no en bloc tissues remained. Lysates were micro-centrifuged at 12,000 rpm for 15 min at 4°C, and supernatants were maintained and stored at -80°C after measuring protein concentrations. Procedures for immunoblot analysis were the same as described previously. Anti-phospho-TRAP 220/MED1 (T1457) antibody (#ab181103, Abcam, UK), anti-TRAP 220/MED1 antibody (#AF 5520-SP, R&D, USA) and anti-α/β-Tubulin antibody (#2148, Cell Signaling Technology, USA) at a dilution of 1:1000 in TBST were used.

### Statistical Analysis

All experiments conducted as part of the *in vitro* cell culture study were repeated three times and those conducted as part of the *in vivo* study were repeated twice, of which one representative result has been included within the supplementary section of the manuscript, since similar results were obtained in all repeats. Statistical Package for the Social Sciences (SPSS) 19.0 software (SPSS Inc., Chicago, IL, USA) was used for analysis and data obtained from multiple independent experiments were expressed as mean ± SD. Statistical differences between the size of tumors before and after treatment was computed using *t* test and other data was computed using Dunnett’s test (for data following normal distribution) or Dunnett's test (for data following non-normal distribution). A *P*-value<0.05 was considered statistically significant.

## Results

### Cell Proliferation Inhibition Rate

**Fig 1:**
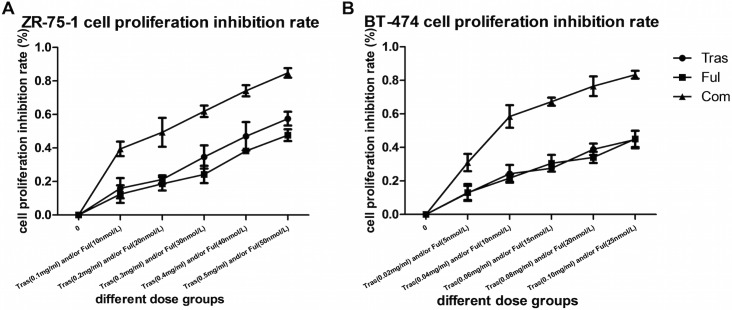
Drug dose versus ZR-75-1 cell proliferation inhibition rate curve (A) and Drug dose versus BT-474 cell proliferation inhibition rate curve (B) using trastuzumab or fulvestrant treatment or both. After 72h of continuous exposure to drugs (either trastuzumab or fulvestrant as single agents) higher cell proliferation inhibition rates were observed with increasing drug concentrations (P<0.05). The combination of both drugs led to much stronger effect compared with individual drug treatments in both cell lines (P<0.05); ED_50_ values have been indicated in Table 1.

Drug dose versus cell proliferation inhibition rate curves for ZR-75-1 cells and BT-474 cells (either untreated or in the presence of trastuzumab or fulvestrant or both) are shown in Fig 1. As no statistical differences between the effects on the vehicle groups and control groups were observed in both cell lines, data from the vehicle group has not been shown. In both cell lines, trastuzumab and fulvestrant as single agents caused an increase in the rate of cell proliferation inhibition with increasing drug concentrations (*P*<0.05), and their combination led to a much stronger effect compared with the use of individual drugs(*P*<0.05).

Analyses of the combined use of trastuzumab and fulvestrant conducted using the CalcuSyn software are shown in Table 1. 50% effective dose (ED_50_) of trastuzumab and fulvestrant in individual drug experiments were applied in subsequent experiments. At ED_50_ levels, the combination index (CI) was <1 and the dose-reduction index (DRI) was >1 in both cell lines.

**Table 1:** Combinatorial use of trastuzumab and fulvestrant (CalcuSyn).

Cell lines	ED_50_				Treatment	Concentration ratio	DRI (ED_50_)^c^		CI (ED_50_)^d^	E
	Individual drugs		Combination							
	Tras (mg/ml)	Ful (nmol/L)	Tras (mg/ml)	Ful (nmol/L)			Tras	Ful		
**ZR-75-1**	0.65	45.12	0.17	16.74	Tras:Ful	16480.71^a^	3.9	2.7	0.629<1	S
**BT-474**	0.13	34.27	0.03	8.54	Tras:Ful	6592.28^b^	3.8	4.0	0.513<1	S

DRI values indicate that the doses of trastuzumab and fulvestrant required in the combination treatment groups are much lower than those used in the individually treated groups in both cell lines. Despite the use of lower concentrations in the combination treatment group, equal effects were observed over the same period as in individually treated groups. Thus, both drugs exhibited synergistic effects when used in combination as CI<1 for both drug combinations and cell lines.

^a^ Tras:Ful concentration ratio = 0.1(g/L)/(606.77×10×10^−9^)(g/L) = 16480.71.

^b^ Tras:Ful concectration ratio = 0.02(g/L)/(606.77×5×10^−9^)(g/L) = 6592.28. (606.77: MW of fulvestrant.

^c^ DRI value at the ED_50_ level.

^d^ CI value at the ED_50_ level. Tras: Trastuzumab; Ful: Fulvestrant; E: Effect; S: Synergism.

### Effects on Cell Apoptosis

**Fig 2:**
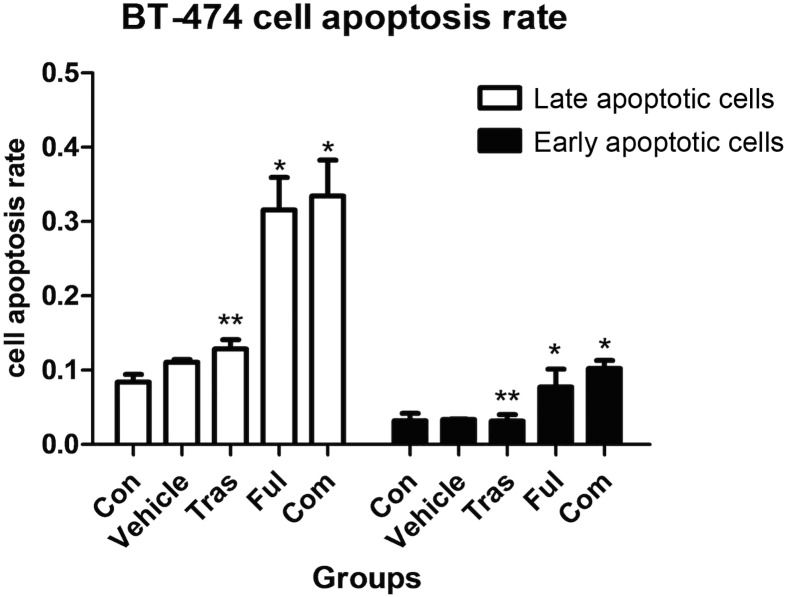
Effects on BT-474 cell apoptosis in either untreated or cultured in the presence of absolute ethanol (vehicle) or trastuzumab at ED_50_ or fulvestrant at ED_50_ or both for 72h. Data are representative of at least three independent experiments with similar results. Fulvestrant as a single agent or combined with trastuzumab induced BT-474 cell apoptosis. However, although significant differences were observed between the trastazumab-treated and combination groups, no statistical differences were observed between the fulvestrant-treated and combination groups. * P<0.05 compared with control group. ** P<0.05 compared with combination group.

**Fig 3:**
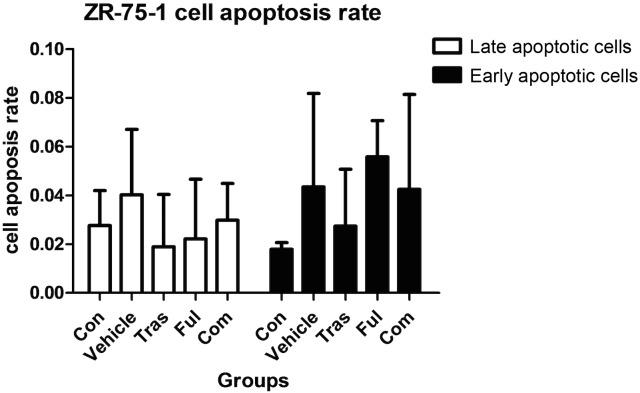
Effects on ZR-75-1 cell apoptosis either untreated or cultured in the presence of the vehicle or trastuzumab at ED_50_ or fulvestrant at ED_50_ or both for 72h. Data are representative of at least three independent experiments with similar results. Statistically significant differences were not observed in the rate of cell apoptosis between treatment groups and control groups in the ZR-75-1 cell line (*P*>0.05).

It was clear that fulvestrant as a single agent or combined with trastuzumab induced both, early stage apoptosis (*P*<0.001 for both) and late stage apoptosis (*P* = 0.013 for the fulvestrant-treated group and *P* = 0.002 for combination group) in BT-474 cells. However, no statistical differences in the rates of cell apoptosis were observed between the two groups receiving fulvestrant as a single agent or in combination with trastuzumab (Fig 2 and Table 2). Besides, no increase in the rate of cellular apoptosis was observed in the trastuzumab-treated group for the BT-474 cell line (Fig 2 and Table 2) and in all the treatment groups for ZR-75-1 cell line (Fig 3 and Table 3).

**Table 2:** Effects on BT-474 cell apoptosis treated with trastuzumab and fulvestrant as single agents or with a combination of both.

Groups	Drug concentration	Early apoptosis rate ($\bar{x}\pm s$)	Late apoptosis rate ($\bar{x}\pm s$)
**Control**	0	0.0840±0.0104	0.0319±0.0099
**Vehicle**	0.0034%^a^	0.1107±0.0035	0.0334±0.0009
**Trastuzumab**	0.13mg/ml	0.1287±0.0123	0.0315±0.0088
**Fulvestrant**	34nmol/L	0.3157±0.0438^b^	0.0772±0.0241^c^
**Combination**	Tras (0.13mg/ml) + Ful (34nmol/L)	0.3343±0.0482^b^^d^	0.1023±0.0110^e^^f^

^a^ cells in the vehicle group were cultured in the presence of the same concentration of absolute ethanol as the fulvestrant-treated group.

^b^ *P*<0.001 compared with control group.

^c^ *P* = 0.013 compared with control group.

^d^ *P*<0.001 compared with trastuzumab-treated group.

^e^ *P* = 0.002 compared with control group.

^f^ *P* = 0.002 compared with trastuzumab-treated group.

**Table 3:** Effects on ZR-75-1 cell apoptosis treated with trastuzumab and fulvestrant as single agents or with a combination of both.

Groups	Drug concentration	Early apoptosis rate ($\bar{x}\pm s$)	Late apoptosis rate ($\bar{x}\pm s$)
**Control**	0	0.0276±0.0144	0.0179±0.0027
**Vehicle**	0.0045%^a^	0.0402±0.0268^b^	0.0435±0.0383^b^
**Trastuzumab**	0.65mg/ml	0.0189±0.0215^b^	0.0274±0.0233^b^
**Fulvestrant**	45nmol/L	0.0221±0.0245^b^	0.0558±0.0148^b^
**Combination**	Tras (0.65mg/ml) + Ful (45nmol/L)	0.0298±0.0151^b^^c^^d^	0.0425±0.0389^b^^c^^d^

^a^ Cells in vehicle group were cultured in the presence of the same concentration of absolute ethanol as fulvestrant-treated group.

^b^ *P*>0.05 compared with control group.

^c^ *P*>0.05 compared with trastuzumab-treated group.

^d^ *P*>0.05 compared with fulvestrant-treated group

### Effects on Cell Cycle Progression

**Fig 4:**
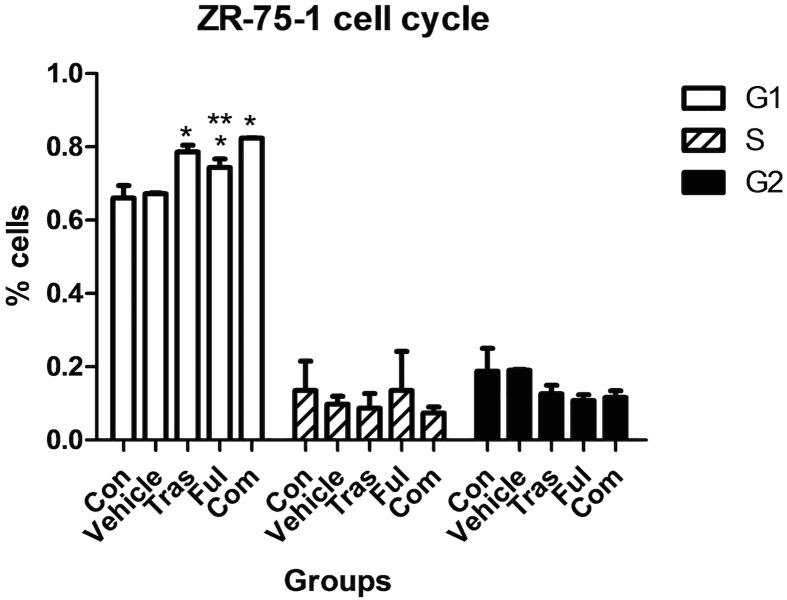
Effects on the cell cycle stage distribution of ZR-75-1 cells after continuous exposure to trastuzumab and fulvestrant as single agents or in combination. Trastuzumab and fulvestrant used as single agents promoted a statistically significant accumulation of ZR-75-1 cells in the G1 phase of the cell cycle when compared to the control groups. The combination of trastuzumab and fulvestrant caused a statistically significant further increase in the percentage of ZR-75-1 cells in the G_1_ phase compared with the effect of fulvestrant alone (*P* = 0.030). * P<0.05 compared to the control group. ** P<0.05 compared to the combination group. The cell cycle stage distribution of ZR-75-1 cells in the five different groups are shown in S3 Fig of the supplementary information section.

**Fig 5:**
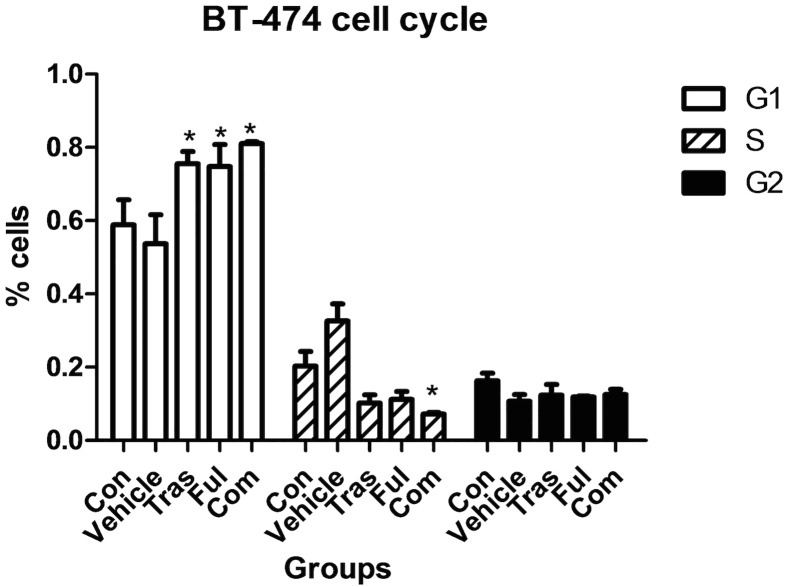
Effects on the cell cycle progression of BT-474 cells after continuous exposure to trastuzumab and fulvestrant as single agents or in combination for 72h. Trastuzumab and fulvestrant as single agents or as a combination promoted the accumulation of BT-474 cells in the G1 phase of the cell cycle. However, no statistically significant differences were detected between the combination treatment group and individual drug-treated groups (P>0.05). The cell cycle stage distribution of BT-474 cells in the five different groups are shown in S4 Fig of the supplementary information section. * P<0.05 compared with control group.

The distribution of ZR-75-1 cells in different phases of the cell cycle after 72h drug treatments is shown in Fig 4 and Table 4, and that of BT-474 cells is shown in Fig 5 and Table 5. In our experiments, the use of trastuzumab and fulvestrant either as single agents or in combination promoted an accumulation of both ZR-75-1 and BT-474 cells in the G_1_ phase of the cell cycle (compared with control group (*P*<0.05)). The combination of trastuzumab and fulvestrant caused a statistically significant further increase in the percentage of ZR-75-1 cells in the G_1_ phase compared with the effect of fulvestrant alone (*P* = 0.030), but no significant differences were observed between the trastuzumab-treated group and the combination group (*P*>0.05). Analysis of the distribution of the BT-474 cells in different phases of the cell cycle indicated that no statistically significant differences were detected between the combination group and individual drug-treatment groups(*P*>0.05).

**Table 4:** Effects on the cell cycle progression of ZR-75-1 cells treated with trastuzumab or fulvestrant or with a combination of both.

Groups	Drug concentration	G_1_ (%, $\bar{x}\pm s$)	S (%, $\bar{x}\pm s$)	G_2_ (%, $\bar{x}\pm s$)
**Control**	0	66.05±3.37	13.53±8.01	18.80±6.26
**Vehicle**	0.0045%^a^	67.19±0.25	9.78±2.15	19.05±0.25
**Trastuzumab**	0.65mg/ml	78.60±1.83^b^	8.67±4.07	12.59±2.34
**Fulvestrant**	45nmol/L	74.38±2.33^c^	13.52±1.68	1.08±1.55
**Combination**	Tras (0.65mg/ml) + Ful (45nmol/L)	82.38±0.07^d^^e^	7.36±1.65	11.59±1.87

^a^ cells in vehicle group were cultured in the presence of the same concentration of absolute ethanol as fulvestrant-treated group.

^b^ *P* = 0.005 compared with control group.

^c^ *P* = 0.026 compared with control group.

^d^ *P* = 0.001 compared with control group.

^e^ *P* = 0.030 compared with fulvestrant-treated group.

**Table 5:** Effects on the cell cycle progression of BT-474 cells treated with trastuzumab or fulvestrant or with a combination of both.

Groups	Drug concentration	G_1_ (%, $\bar{x}\pm s$)	S (%, $\bar{x}\pm s$)	G_2_ (%, $\bar{x}\pm s$)
**Control**	0	58.91±6.80	20.33±3.96	16.32±2.03
**Vehicle**	0.0034%^a^	53.71±7.91	32.61±4.72	10.68±1.90
**Trastuzumab**	0.13mg/ml	75.54±3.30^b^	10.17±2.30	12.34±2.98
**Fulvestrant**	34nmol/L	74.82±5.97^c^	11.24±2.15	11.85±0.25
**Combination**	Tras (0.13mg/ml) + Ful (34nmol/L)	81.07±0.47^d^	7.17±0.47^e^	12.53±1.43

^a^ cells in vehicle group were cultured in the presence of the same concentration of absolute ethanol as fulvestrant-treated group.

^b^ *P* = 0.004 compared with control group.

^c^ *P* = 0.005 compared with control group.

^d^ *P* = 0.002 compared with control group.

^e^ *P* = 0.030 compared with control group.

### Immunoblot Analysis of Downstream Signaling Pathways

**Fig 6:**
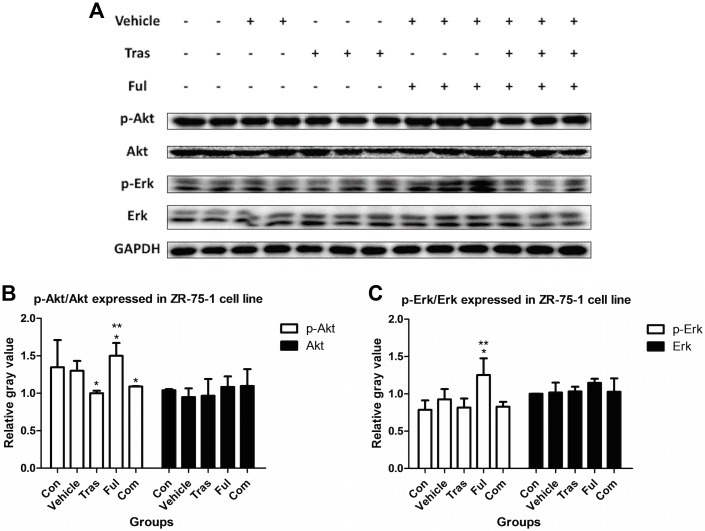
Effects on the levels of the proteins p-Akt and p-Erk extracted from ZR-75-1 cells either untreated or cultured in the presence of vehicle or trastuzumab at ED_50_ or fulvestrant at ED_50_ or with a combination of both for 72h. p-Akt and p-Erk proteins from two samples in the control group and vehicle group and from three samples in each treatment group were detected. It was found that trastuzumab decreased p-Akt levels and that fulvestrant increased both, p-Akt and p-Erk levels. In addition, the combination of both drugs significantly reduced levels of p-Akt and notably inhibited the effect that fulvestrant had on the levels of both proteins. * P<0.05 compared with control group. ** P<0.05 compared with combination group.

**Fig 7:**
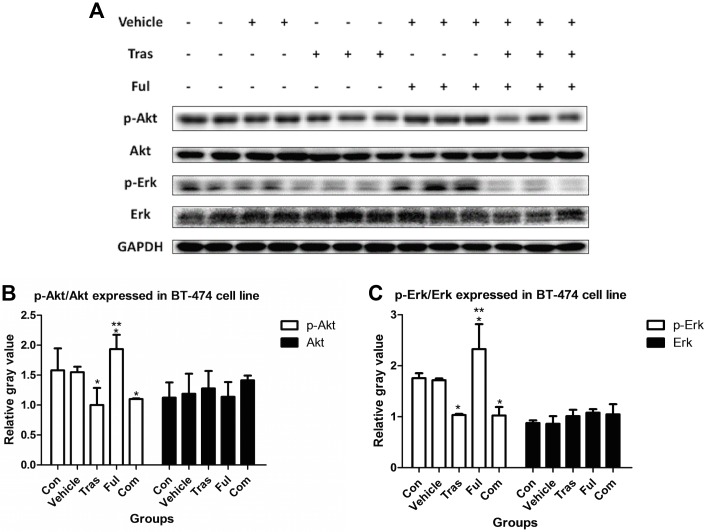
Effects on the levels of the proteins p-Akt and p-Erk extracted from BT-474 cells either untreated or cultured in the presence of vehicle or trastuzumab at ED_50_ or fulvestrant at ED_50_ or with a combination of both for 72h. The figure shows that trastuzumab when used as a single agent decreased the levels of both p-Akt and p-Erk, while fulvestrant raised the levels of both proteins. The combination of both drugs significantly prevented fulvestrant from raising the levels of both proteins. * P<0.05 compared with control group. ** P<0.05 compared with combination group.

In work with proteins extracted from ZR-75-1 cells (Fig 6), levels of the p-Akt protein decreased in the trastuzumab-treated group (Tras vs. Con, *P* = 0.037) and the levels of p-Akt and p-Erk increased in the fulvestrant-treated group (Ful vs. Con, *P* = 0.005 for p-Akt and *P* = 0.025 for p-Erk). The combination of trastuzumab and fulvestrant significantly reduced p-Akt protein levels (Com vs. Con, *P* = 0.020) and notably also inhibited the effect of fulvestrant on the increased levels of p-Akt and p-Erk (Com vs. Ful, *P* = 0.046 for p-Akt and *P* = 0.025 for p-Erk). For samples extracted form BT-474 cells (Fig 7), trastuzumab as a single agent or in combination with fulvestrant reduced the levels of p-Akt and p-Erk proteins (Tras vs. Con, *P* = 0.029 for p-Akt and *P* = 0.033 for p-Erk; Com vs. Con, *P* = 0.019 for p-Akt and *P* = 0.038 for p-Erk). Fulvestrant, however, raised protein expression levels (Ful vs. Con, *P* = 0.015 for p-Akt and *P* = 0.047 for p-Erk). The combination of trastuzumab and fulvestrant significantly inhibited the effects of fulvestrant on protein expression levels (Com vs. Ful, *P* = 0.001 for p-Akt and p-Erk).

### Studies Using a Mouse Tumor Xenograft Model

**Fig 8:**
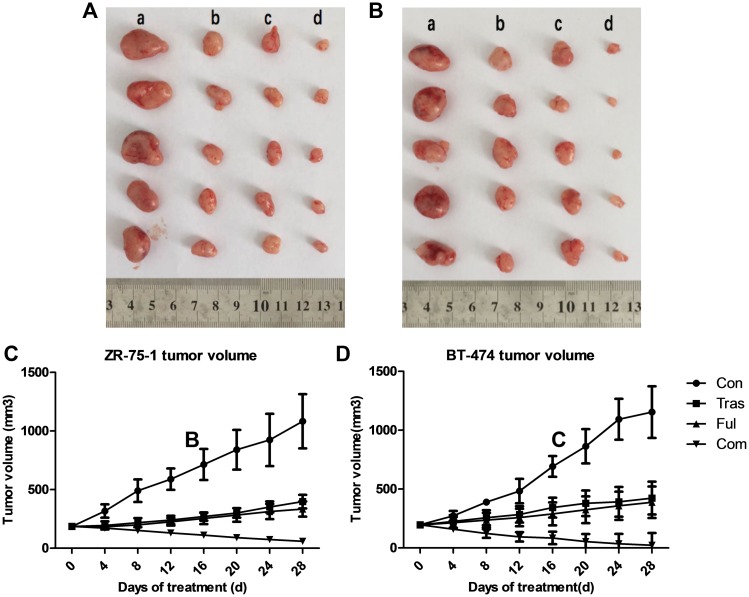
Nude Mouse Xenograft Model Studies. ZR-75-1 (a) and BT-474 (b) tumor xenografts excised from female BALB/c (nu/nu) nude mice after four-week treatments with trastuzumab and fulvestrant as single agents or in combination. Days versus tumor volume curves for ZR-75-1 (c) and BT-474 (d) tumor xenografts show that trastuzumab and fulvestrant as single agents or in combination significantly inhibited tumor growth (*P*<0.05) and that tumor growth in the combination group was notably much more inhibited compared with that in individually treated groups (*P*<0.05).

Nude mice bearing tumor xenografts between 150-250mm^3^ in size were randomly assigned to vehicle-treated, trastuzumab-treated, fulvestrant-treated or combination treatment groups. 4 weeks post treatment, tumors were excised (ZR-75-1 tumor xenografts are shown in Fig 8a and BT-474 tumor xenografts in Fig 8b) and stored at -80°C for immunoblot analysis. Tumor growth curves of ZR-75-1 and BT-474 tumor xenografts are shown in Fig 8c and 8d respectively. The figures indicate that both ZR-75-1 and BT-474 tumors in trastuzumab-treated and fulvestrant-treated groups increased in size after the 4-week treatment period (*P*<0.001, *t* test), but were still significantly smaller than those in the vehicular control group (*P*<0.001, Dunnett’s test). However, the combination of trastuzumab and fulvestrant, resulted in the greatest inhibition of tumor growth compared to either drug alone (*P*<0.001, Dunnett’s test), shrinking tumor size significantly at the termination of the experiment (*P*<0.001, *t* test). Moreover, it is also noteworthy that BT-474 tumor xenografts were so sensitive to the combination treatment that several tumors in the combination treatment group almost disappeared after four-week treatment (with much lower drug doses than those used for mice bearing ZR-75-1 tumor xenografts), leaving a cavity in place of the xenografts. In addition, weight change curves of nude mice in ZR-75-1 (S5A Fig) and BT-474 (S5B Fig) tumor xenograft models showed that the weight of the mice in both animal models in all groups did not change significantly through the entire treatment process (*P*>0.05).

### Immunoblot Analysis of Proteins Expressed in Tumor Tissues

**Fig 9:**
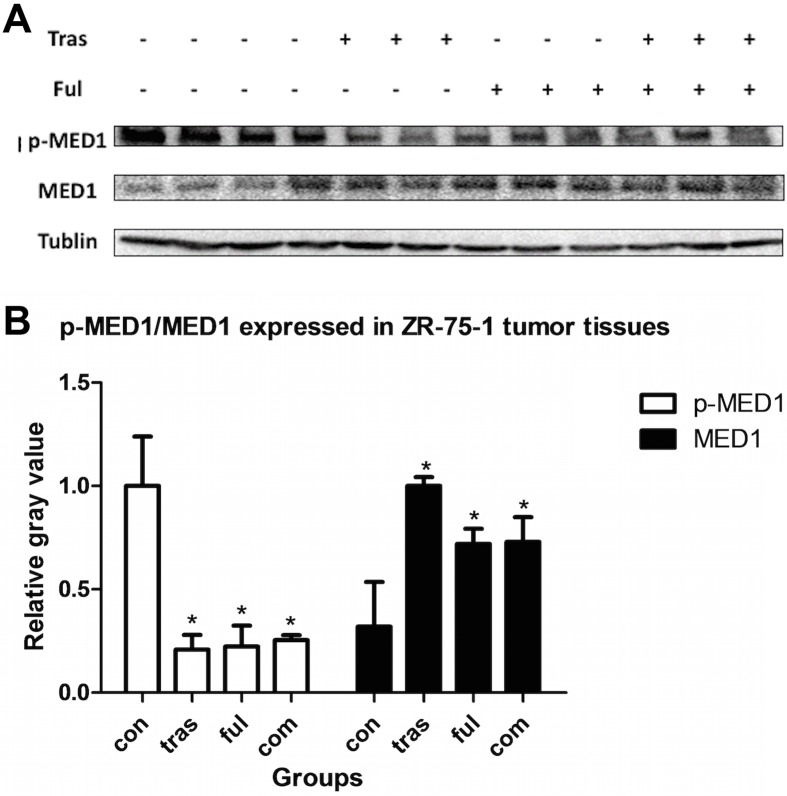
Immunoblot analysis of the levels of the proteins p-MED1 and MED1 expressed in ZR-75-1 tumor xenografts. Three samples from each group were randomly selected. Similar results were obtained after two experimental repeats. Thus, representative bands from a single experiment are shown here. Trastuzumab and fulvestrant either as single agents or in combination decreased levels of the protein p-MED1 (*P*<0.05) and increased levels of MED1 total protein (*P*<0.05). However, no significant differences were observed between combination groups and individually treated groups (*P*>0.05).

**Fig 10:**
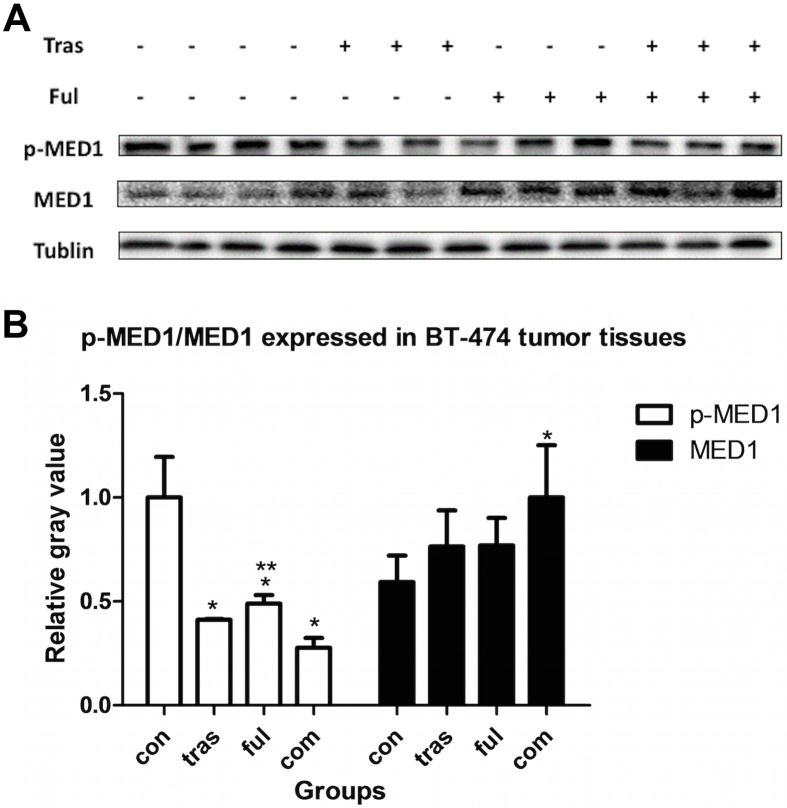
Immunoblot analysis of the levels of the proteins p-MED1 and MED1 expressed in BT-474 tumor xenografts. Three samples from each group were randomly selected. Similar results were obtained after two experimental repeats. Thus, representative bands from a single experiment are shown here. Trastuzumab and fulvestrant as single agents decreased levels of the protein p-MED1 (*P*<0.05) and the combination resulted in lower expression levels when compared with fulvestrant alone (*P* = 0.039). In addition, total protein levels of MED1 increased in the combination group compared with the vehicle-treated group (*P* = 0.017).

Since levels of the protein p-MED1 (the phosphorylated form of the Mediator Complex Subunit 1) detected within the two cell lines were too low to allow the quantification of any variations caused due to drug treatment, we analyzed this parameter in mouse tumor xenograft tissues using immunobloting. As shown in Fig 9, trastuzumab and fulvestrant, either as single agents or in combination decreased the levels of the p-MED1 (phosphorylated MED1) protein expressed in ZR-75-1 tumor xenografts (*P*<0.001 for all the treatment groups compared with vehicle-treated group), and simultaneously increased expression levels of total MED1 protein (Tras vs. vehicle, *P*<0.001; Ful vs. vehicle, *P* = 0.011; Com vs. vehicle, *P* = 0.010). However, no statistically significant differences in both p-MED1 and MED1 protein expression levels were observed between the combination and individually treated groups (*P*>0.05). Results from the BT-474 tumor xenograft model shown in Fig 10 indicate that the level of protein p-MED1 in all treatment groups decreased compared with vehicle-treated group (*P*<0.001), and that the combination group expressed lower levels of p-MED1 than that the fulvestrant-treated group (*P* = 0.039). Besides, trastuzumab combined with fulvestrant could enhance the expression of total protein MED1 (*P* = 0.017). Therefore, it is possible that trastuzumab acts in synergy with fulvestrant in HR+/HER2+ breast cancer by suppressing the activation of the protein MED1.

## Discussion

Our results highlight the effects of trastuzumab (herceptin) and fulvestrant (falsodex) as single agents or in combination on the cell proliferation rates of HR+/HER2+ human breast cancer cell lines ZR-75-1 and BT-474. We show that these effects are concentration-dependent, and that treatment with a combination of both drugs results in a synergistic inhibition of cell growth in culture. This demonstration of synergy between trastuzumab and fulvestrant on human cancer cell lines has not been reported previously. Besides, the doses of both drugs decrease in the combined application (with DRI>1 at ED_50_), increasing the likelihood that the occurrence of side effects will reduce when used in combination physiologically. Further experiments that were conducted as part of this work sought to investigate the basis and extent of this synergistic effect and thus experiments on cell apoptosis, cell cycle progression and downstream cell signaling pathways were carried out.

An analysis of drug effects on the rate of cell apoptosis using the Annexin-V/PI assay indicated that fulvestrant, either as a single agent or in combination with trastuzumab promotes BT-474 cell apoptosis. However, no significant differences in apoptotic rate between the fulvestrant-treated group and the combination groups were observed. In addition, no effect on the rate of BT-474 cell apoptosis was observed upon the administration of trastuzumab alone. Therefore, in this case, the combination of trastuzumab and fulvestrant did not have a synergistic, pro-apoptotic effect and any increase in the rate of BT-474 apoptosis observed could be attributed to the use of fulvestrant alone. Moreover, neither drug was capable of inducing apoptosis in the ZR-75-1 cell line. Thus, although both cell lines used were HR+/HER2+, they responded differently to therapy in our experiments. This is an important consideration even during the clinical management of patients and further highlights the heterogenous nature of triple positive breast cancer.

Previous studies have reported that when trastuzumab and fulvestrant are used as single agents, they promote an accumulation of cells in the G1 phase of the cell cycle[44] and we confirmed these results in our experiments on both cell lines. In addition, an increased effect on the accumulation of cells in the G1 phase was observed in the combination group compared with the fulvestrant-treated group in the ZR-75-1 cell line. However, in our experiments on the BT-474 cell line, no significant differences were observed between combination and individually treated groups.

Several previous studies have documented that trastuzumab inhibits the activation of the downstream signaling pathways involving PI3K/Akt/mTOR and MAPK/Erk[45,46], and we report similar results in our work. We observed that trastuzumab treatment decreased levels of the protein p-Akt in both cell lines and also decreased levels of the protein p-Erk in BT-474 cells. Besides, both p-Akt and p-Erk were found to be overexpressed in ZR-75-1 as well as BT-474 cells after fulvestrant treatment but notably decreased from these levels when combined with trastuzumab, implying that the combination of trastuzumab and fulvestrant significantly prevented fulvestrant from increasing the levels of these proteins in HR+/HER2+ breast cancer. Since the activation of PI3K/Akt/mTOR and MAPK/Erk signaling pathways correlates with worse clinical outcomes in breast cancer[47,48], our results suggest that HR+/HER2+ breast cancer patients receiving co-targeted therapies including trastuzumab and fulvestrant in combination could probably have a better prognosis than those receiving fulvestrant alone.

Consistent with the results from *in vitro* experiments reported in this work, *in vivo* studies using ZR-75-1 and BT-474 tumor xenografts showed that both, trastuzumab and fulvestrant as single agents inhibited tumor growth. In addition, their combination resulted in an enhanced inhibition compared to either drug alone and apart from preventing further tumor growth, even effectively shrank pre-existing tumors. Moreover, it is noteworthy that the BT-474 tumor xenografts were extremely sensitive to the combination of trastuzumab and fulvestrant and we show that several tumors in the combination treatment group almost disappeared after a four-week long treatment with a much lower drug concentration than was used for mice bearing ZR-75-1 tumor xenografts. Hence, the combination of trastuzumab and fulvestrant is synergistic and effectively inhibits HR+/HER2+ breast cancer tumor growth *in vivo*.

Using ZR-75-1 and BT-474 mouse tumor xenograft models, we showed that trastuzumab and fulvestrant as single agents or in combination could also significantly inhibit the activation of MED1 and that their combination resulted in a much stronger inhibition than fulvestrant alone in work with BT-474 tumor xenograft model. This difference in response to the two drugs can most likely be attributed to the fact that the BT-474 cell line is known to express much higher levels of HER2, which is the molecular target of trastuzumab[49]. However, in our work with the ZR-75-1 tumor xenograft model, no significant differences were observed between the combination group and single agent-treated groups. This was probably due to the fact that the initial doses of trastuzumab and fulvestrant used were already saturating. The lack of difference observed between individual and combined treatment groups could also be attributed to the lengthy duration of treatment. Importantly, the activation of MED1 has been shown to correlate with poor clinical outcomes in breast cancer patients treated with endocrine therapy[33] and our results indicate that trastuzumab and fulvestrant may improve the prognosis of HR+/HER2+ breast cancer patients by inhibiting the activation of MED1.

Fulvestrant, a novel endocrine modulator, has been reported to remain active even after recurrence or progression with previous endocrine treatments in postmenopausal women[40–42,45]. Therefore, the combination of trastuzumab and fulvestrant, which had positive synergistic effects in our experiments, may constitute an improved course of treatment for HR+/HER2+ advanced breast cancer resistant to previous hormone therapies.

## Supporting Information

S1 Fig Flow cytometry results showing the effects of different treatments on BT-474 cell apoptosis. (TIF)

S2 Fig Flow cytometry results showing the effects of different treatments on ZR-75-1 cell apoptosis. (TIFF)

S3 Fig The cell cycle stage distribution of ZR-75-1 cells in the five different groups. (TIFF)

S4 Fig The cell cycle stage distribution of BT-474 cells in the five different groups. (TIFF)

S5 Fig Weight change curves of nude mice in ZR-75-1 (S5A) and BT-474 (S5B) tumor xenograft models. (TIF)
