# Correction: An Experimental Analysis of the Molecular Effects of Trastuzumab (Herceptin) and Fulvestrant (Falsodex), as Single Agents or in Combination, on Human HR+/HER2+ Breast Cancer Cell Lines and Mouse Tumor Xenografts

**DOI:** 10.1371/journal.pone.0311128

**Published:** 2024-09-23

**Authors:** Qing Chen, Ziyi Weng, Yunshu Lu, Yijun Jia, Longlong Ding, Fang Bai, Meixin Ge, Qing Lin, Kejin Wu

After publication of this article [1], concerns were raised about similarity with a closely related article by the same authors in the *Chinese Journal of Practical Surgery* [2] which was not discussed in the above publication.

The Corresponding author (KW) and first author (QC) responded to the concern, stating that the results presented in [2] are from preliminary experiments investigating the effects of 0.5g/L or 1.0g/L trastuzumab and 15nmol/L fulvestrant on human HR+/HER2+ breast cancer cell line BT-474 for 48h, including effects on cell proliferation, cell apoptosis, and the expression levels of proteins p-Akt and p-MAPK, whereas the *PLOS ONE* article [1] presents the results of follow-up experiments, which investigated the effects of 0.13mg/ml trastuzumab and 34nmol/L fulvestrant on a BT-474 cell line, and of 0.65mg/ml trastuzumab and 45nmol/L fulvestrant on a ZR-75-1 cell line, both for 72h. Furthermore, in the follow-up experiments presented in [1], the authors also studied these effects in mouse tumor xenografts.

Additionally, in contrast to the Data Availability statement in [1], all relevant data were not provided within the article and its Supporting Information files. With this Correction, the authors provide the original raw data in S1–S2 Files and via Dryad: https://doi.org/10.5061/dryad.7mp37.

The Data Availability statement is updated to: All relevant data are within the paper, its Supporting Information files, and via Dryad: https://doi.org/10.5061/dryad.7mp37.

A member of the *PLOS ONE* Editorial Board reviewed the concerns and was satisfied that the two articles [1, 2] are sufficiently distinct from one another to warrant separate publications despite their similar design.

## Supporting information

S1 File Individual-level quantitative data underlying the graphs in Figs 1, 6, 7, 9, and 10. (ZIP)

S2 File Individual-level tumor data underlying Fig 8. (ZIP)

## References

[1] Chen Q, Weng Z, Lu Y, Jia Y, Ding L, Bai F, et al. (2017) An Experimental Analysis of the Molecular Effects of Trastuzumab (Herceptin) and Fulvestrant (Falsodex), as Single Agents or in Combination, on Human HR+/HER2+ Breast Cancer Cell Lines and Mouse Tumor Xenografts. PLoS ONE 12(1): e0168960. 10.1371/journal.pone.0168960 28045951 PMC5207527

[2] Chen Q, Wu K, Ding L, Department of General Surgery, Xin Hua Hospital Affiliated to Shanghai Jiao Tong University School of Medicine (2015). Research on the effectiveness of fulvestrant combined with trastuzumab on the human breast cancer cell line BT-474. Chinese Journal of Practical Surgery 7: 749–752.

