# Supplementary figures and images for: An Experimental Analysis of the Molecular Effects of Trastuzumab (Herceptin) and Fulvestrant (Falsodex), as Single Agents or in Combination, on Human HR+/HER2+ Breast Cancer Cell Lines and Mouse Tumor Xenografts
Source: PLoS One. 2017 Jan 3;12(1):e0168960. doi: 10.1371/journal.pone.0168960 (PMC5207527; doi:10.1371/journal.pone.0168960)

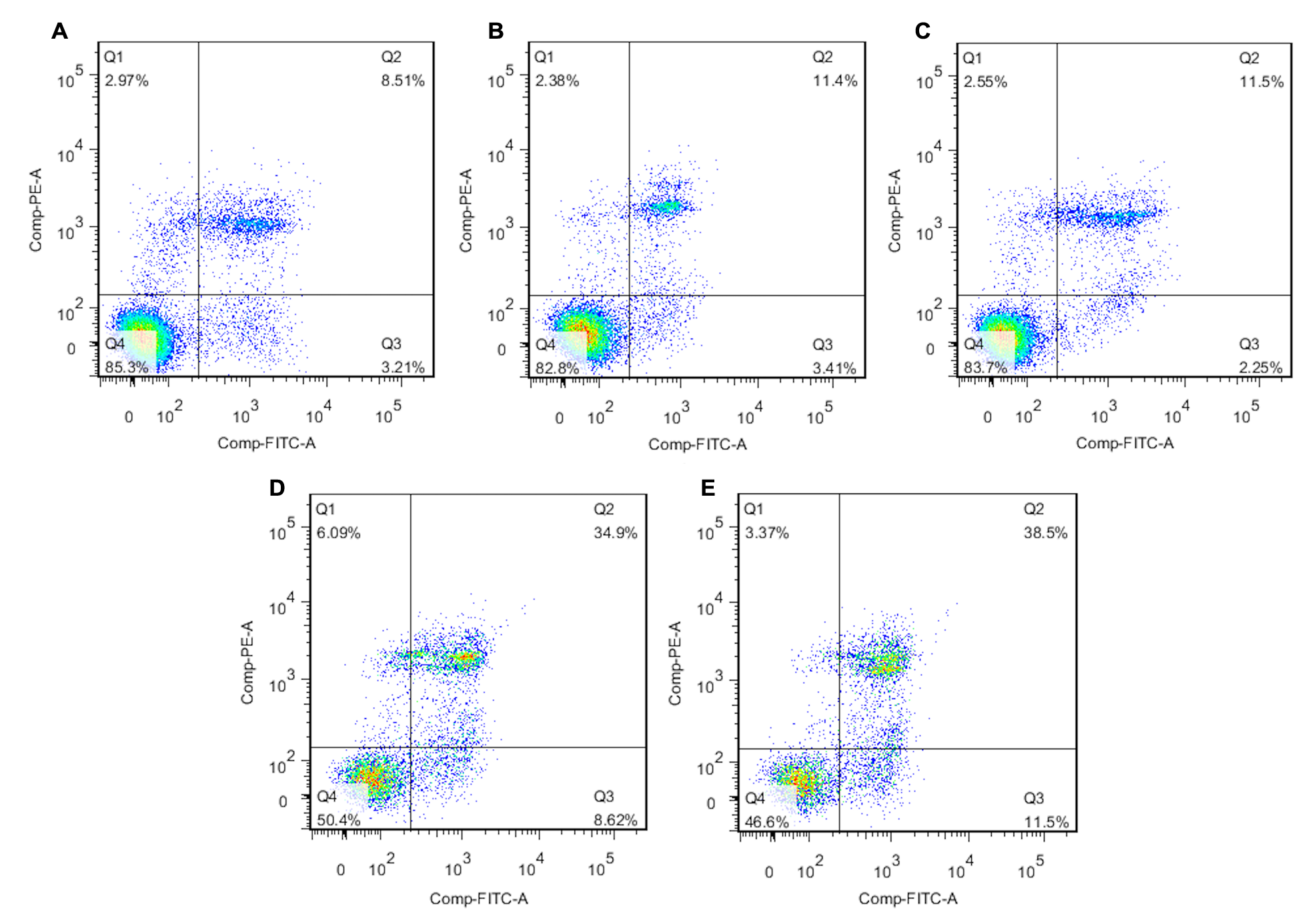

Supplement: S1 Fig — (TIF) [file pone.0168960.s001.tif]

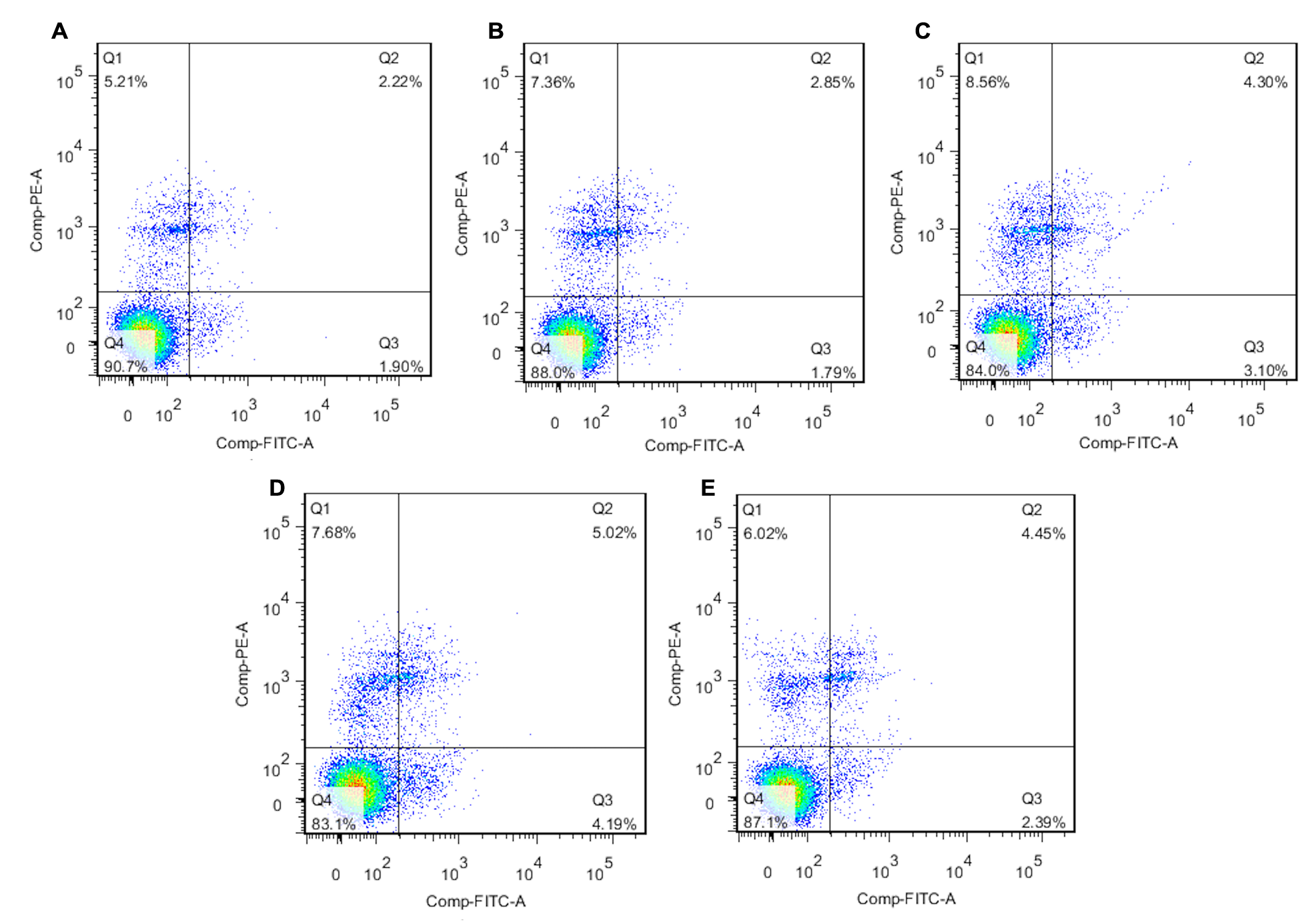

Supplement: S2 Fig — (TIFF) [file pone.0168960.s002.tiff]

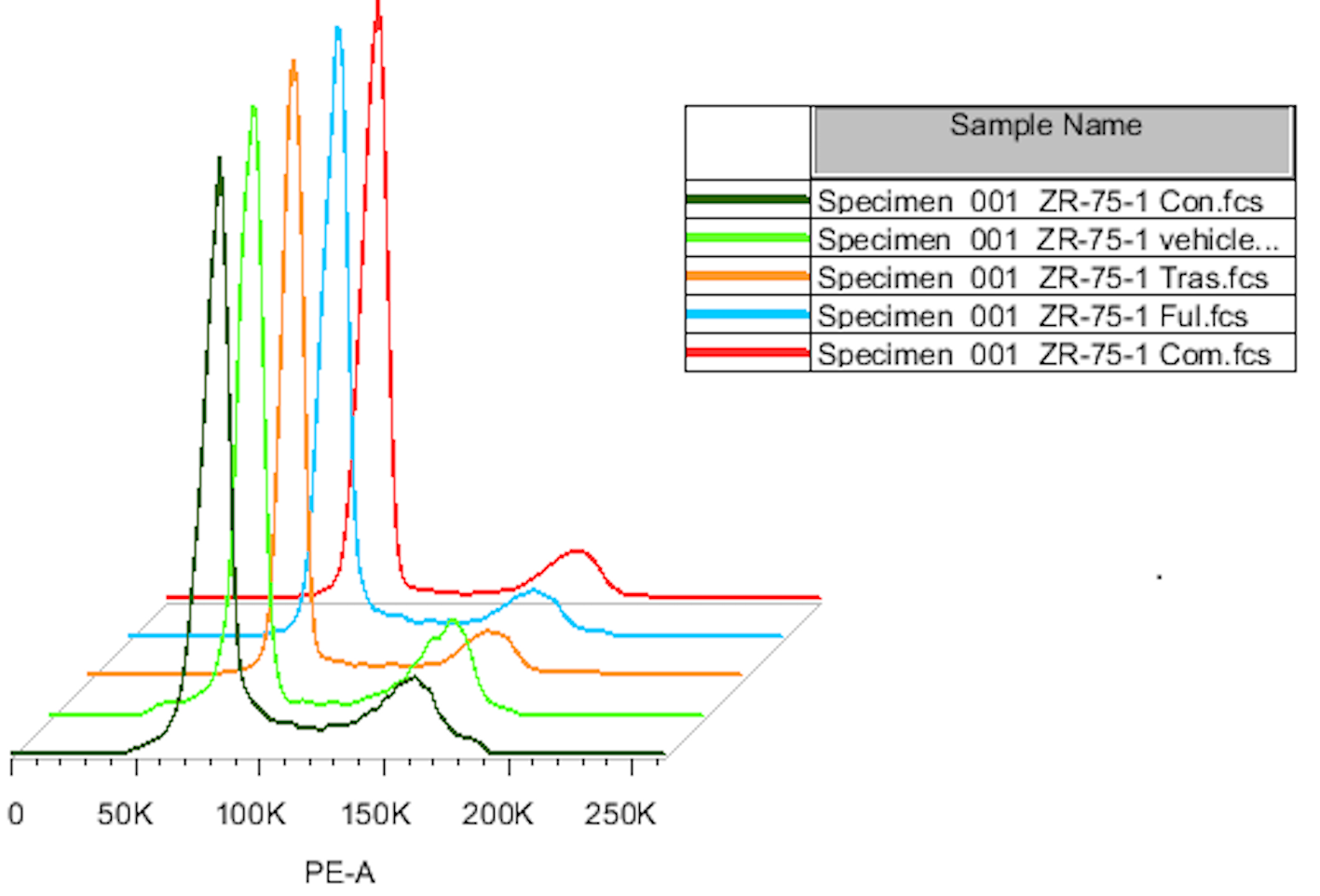

Supplement: S3 Fig — (TIFF) [file pone.0168960.s003.tiff]

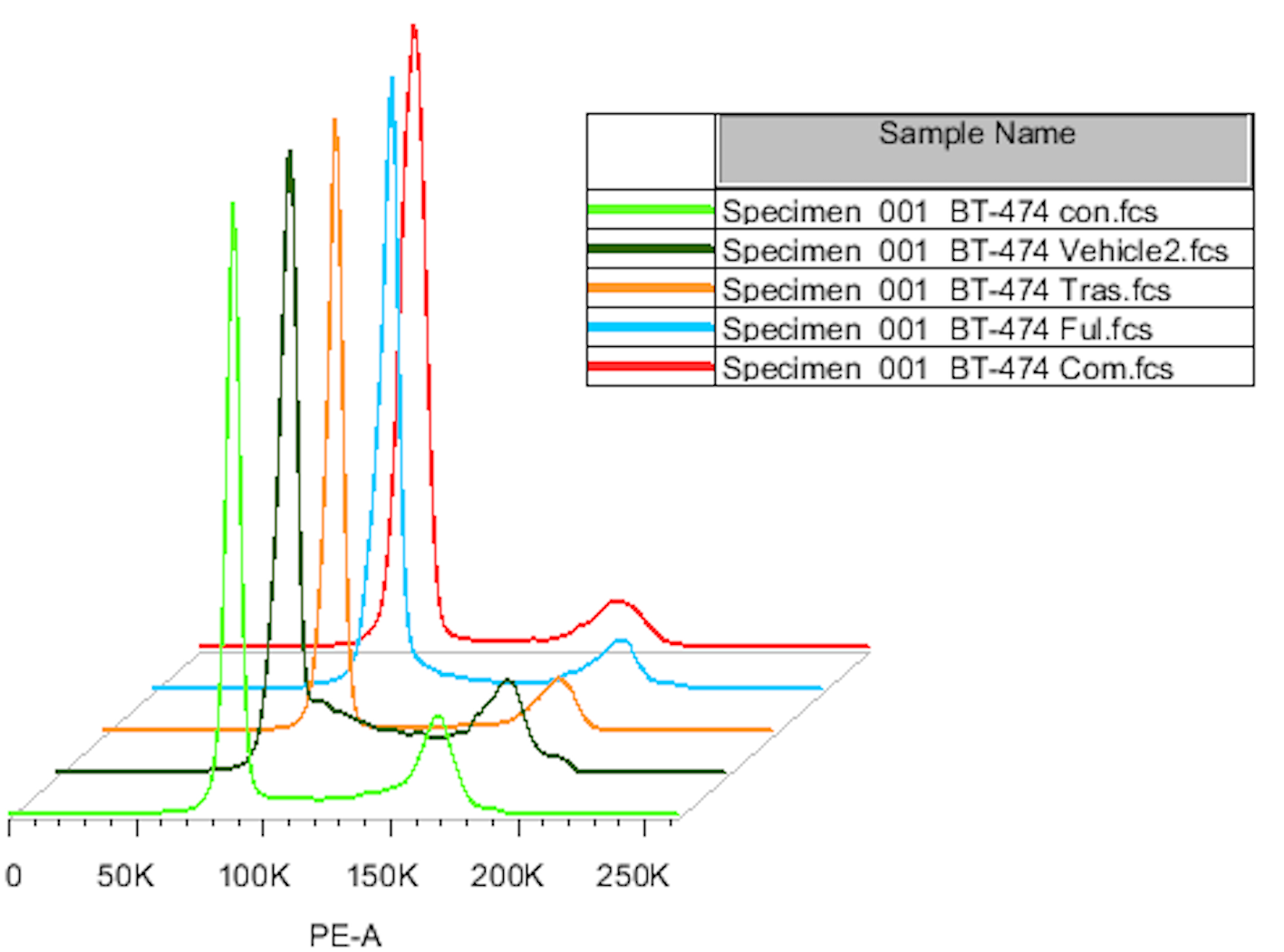

Supplement: S4 Fig — (TIFF) [file pone.0168960.s004.tiff]

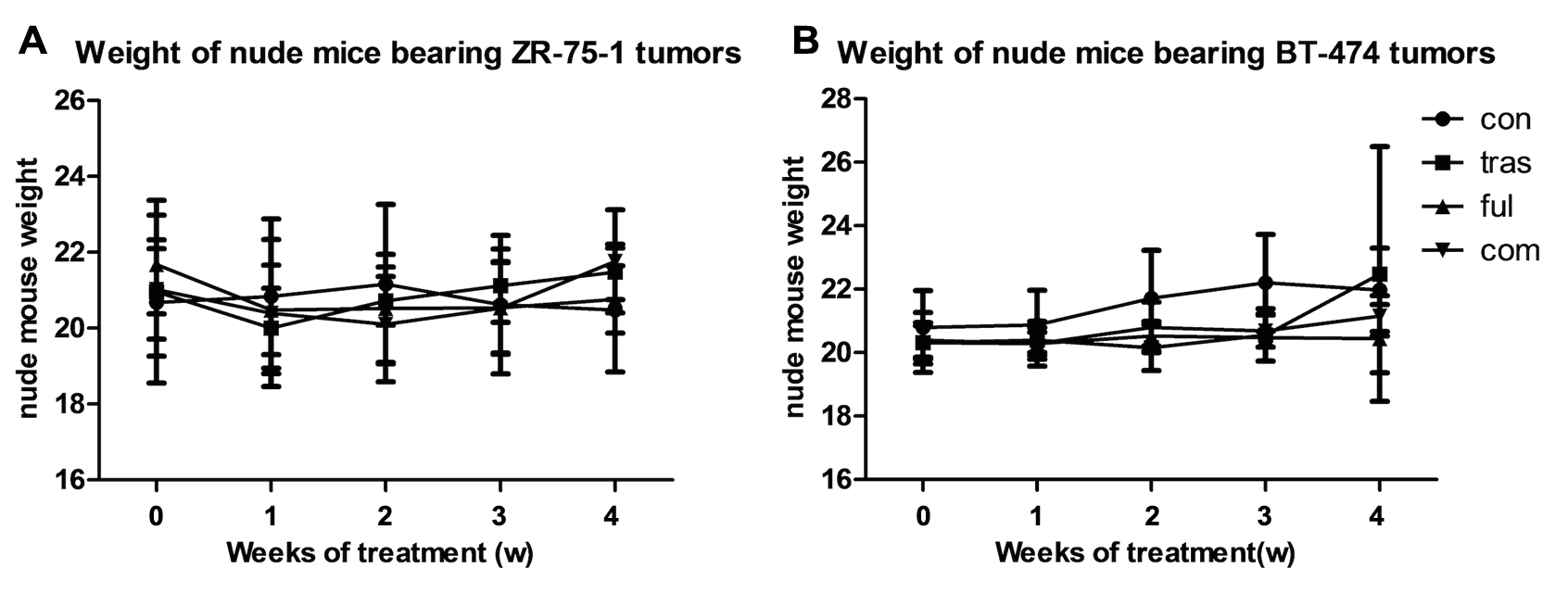

Supplement: S5 Fig — (TIF) [file pone.0168960.s005.tif]
