# Supplementary figures and images for: Correction: An Experimental Analysis of the Molecular Effects of Trastuzumab (Herceptin) and Fulvestrant (Falsodex), as Single Agents or in Combination, on Human HR+/HER2+ Breast Cancer Cell Lines and Mouse Tumor Xenografts
Source: PLoS One. 2024 Sep 23;19(9):e0311128. doi: 10.1371/journal.pone.0311128 (PMC11419337; doi:10.1371/journal.pone.0311128)

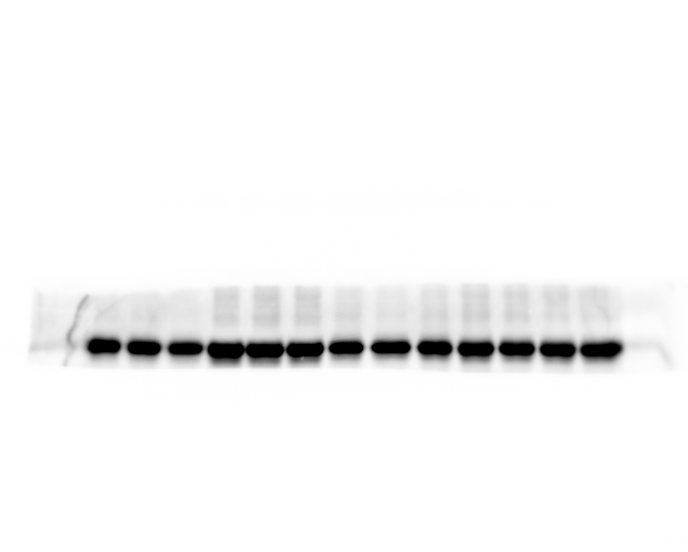

Supplement: S1 File — (ZIP) [file pone.0311128.s001.zip › Figure 6 ZR-75-1 P-AKT.tif]

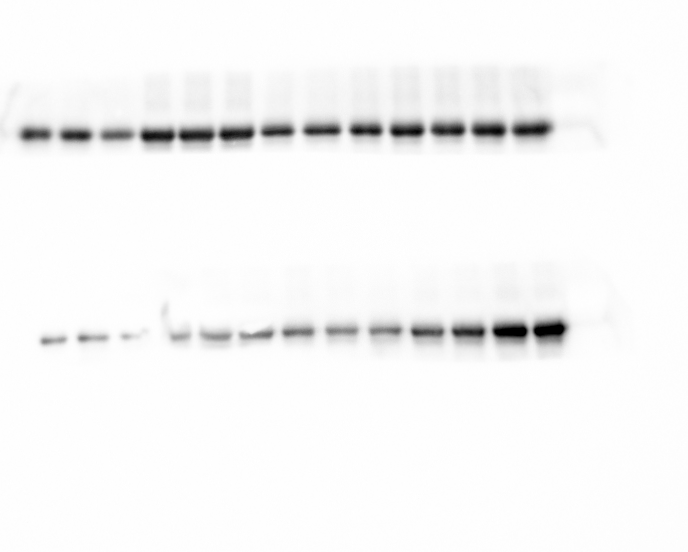

Supplement: S1 File — (ZIP) [file pone.0311128.s001.zip › Figure 7 BT-474 P-AKT.tif]
